# Gut microbe-derived betulinic acid alleviates sepsis-induced acute liver injury by inhibiting macrophage NLRP3 inflammasome in mice

**DOI:** 10.1128/mbio.03020-24

**Published:** 2025-01-31

**Authors:** Xuheng Tang, Tairan Zeng, Wenyan Deng, Wanning Zhao, Yanan Liu, Qiaobing Huang, Yiyu Deng, Weidang Xie, Wei Huang

**Affiliations:** 1 Department of Critical Care Medicine, Guangdong Provincial People’s Hospital, Guangdong Academy of Medical Sciences, Guangzhou, Guangdong, China; 2 Guangdong Provincial Key Laboratory of Cardiac Function and Microcirculation, Department of Pathophysiology, School of Basic Medical Sciences, Southern Medical University, Guangzhou, Guangdong, China; 3 Department of Critical Care Medicine, Nanfang Hospital of Southern Medical University, Guangzhou, Guangdong, China; Catholic University of America, Washington, DC, USA; Memorial Sloan Kettering Cancer Center, New York, New York, USA

**Keywords:** sepsis, acute liver injury, exercise training, gut microbiota, betulinic acid

## Abstract

Sepsis-induced acute liver injury (SALI) is a prevalent and life-threatening complication associated with sepsis. The gut microbiota plays a crucial role in the maintenance of health and the development of diseases. The impact of physical exercise on gut microbiota modulation has been well-documented. However, the potential impact of gut microbiome on exercise training-induced protection against SALI remains uncertain. Here, we discovered exercise training ameliorated SALI and systemic inflammation in septic mice. Notably, gut microbiota pre-depletion abolished the protective effects of exercise training in SALI mice. Fecal microbiota transplantation treatment revealed that exercise training-associated gut microbiota contributed to the beneficial effect of exercise training on SALI. Exercise training modulated the metabolism of *Ligilactobacillus* and enriched betulinic acid (BA) levels in mice. Functionally, BA treatment conferred protection against SALI by inhibiting the hepatic inflammatory response in mice. BA bound and inactivated hnRNPA2B1, thus suppressing NLRP3 inflammasome activation in macrophages. Collectively, this study reveals gut microbiota is involved in the protective effects of exercise training against SALI, and gut microbiota-derived BA inhibits the hepatic inflammatory response via the hnRNPA2B1-NLRP3 axis, providing a potential therapeutic strategy for SALI.

**IMPORTANCE:**

Sepsis is characterized by a dysregulated immune response to an infection that leads to multiple organ dysfunction. The occurrence of acute liver injury is frequently observed during the initial stage of sepsis and is directly linked to mortality in the intensive care unit. The preventive effect of physical exercise on SALI is well recognized, yet the underlying mechanism remains poorly elucidated. Exercise training alters the gut microbiome in mice, increasing the abundance of Ligilactobacillus and promoting the generation of BA. Additionally, BA supplementation can suppress the NLRP3 inflammasome activation in macrophages by directly binding to hnRNPA2B1, thereby mitigating SALI. These results highlight the beneficial role of gut microbiota-derived BA in inhibiting the hepatic inflammatory response, which represents a crucial stride toward implementing microbiome-based therapeutic strategies for the clinical management of sepsis.

## INTRODUCTION

Sepsis is a complex and highly heterogeneous clinical syndrome resulting from an aberrant host response to infection, leading to the development of multiple organ dysfunction syndrome (MODS) (1, 2). Sepsis remains one of the leading causes of global mortality, with an annual incidence of 31.5 million cases and 19.4 million cases of severe sepsis (3). The occurrence of sepsis-induced acute liver injury (SALI) is commonly observed in individuals experiencing septic shock, significantly impacting patient prognosis by increasing the fatality rate to approximately 70%–90% (4). Therefore, the development of novel targeted therapeutic strategies for the prevention and treatment of SALI is urgently needed.

The gut microbiome is crucial in regulating various aspects of human health and illness (5). Emerging evidence indicates that gut microbiome plays a critical role in maintaining host physiology and homeostasis by facilitating the exchange of metabolites and co-metabolizes substrate (6). Disruption of gut microbiota balance has been observed in animal models and patients with SALI (7, 8). Strategies focused on modifying the gut microbiome have been employed to prevent and manage SALI. The regulation of the gut microbiome is a well-known advantage of engaging in exercise training, which contributes positively to human health (9, 10). Furthermore, sepsis can be influenced by exercise training in terms of regulating the balance between inflammation and anti-inflammation, as well as oxidative stress and antioxidative capacity (11, 12). These findings raise the question as to how the gut microbiome facilitates the protective effects of exercise training on SALI. Furthermore, elucidating the molecular mechanism underlying these protective effects of exercise training can contribute to the identification of more potent strategies for the prevention and treatment of sepsis.

In this study, we aimed to investigate the potential roles of the gut microbiome in mediating the protective effects of exercise training against SALI in mice. Using integrative multiomics, we demonstrated that exercise training effectively prevented SALI by modulating the composition and functionality of the gut microbiome. Importantly, exercise training modulated the metabolism of gut microbiome and enhanced production of betulinic acid (BA) in mice. BA was identified as the pivotal metabolite accountable for the protective role of exercise training in sepsis. The protective role of BA as an NLRP3 inflammasome inhibitor in SALI was subsequently established, and its potential therapeutic effect on sepsis was highlighted. Our findings offer novel insights into the protective effect of exercise training against SALI by modulating gut microbiota, in which gut microbe-derived BA plays a pivotal role.

## RESULTS

### Gut microbiota is involved in the protective effects of exercise training in SALI

**Fig 1:**
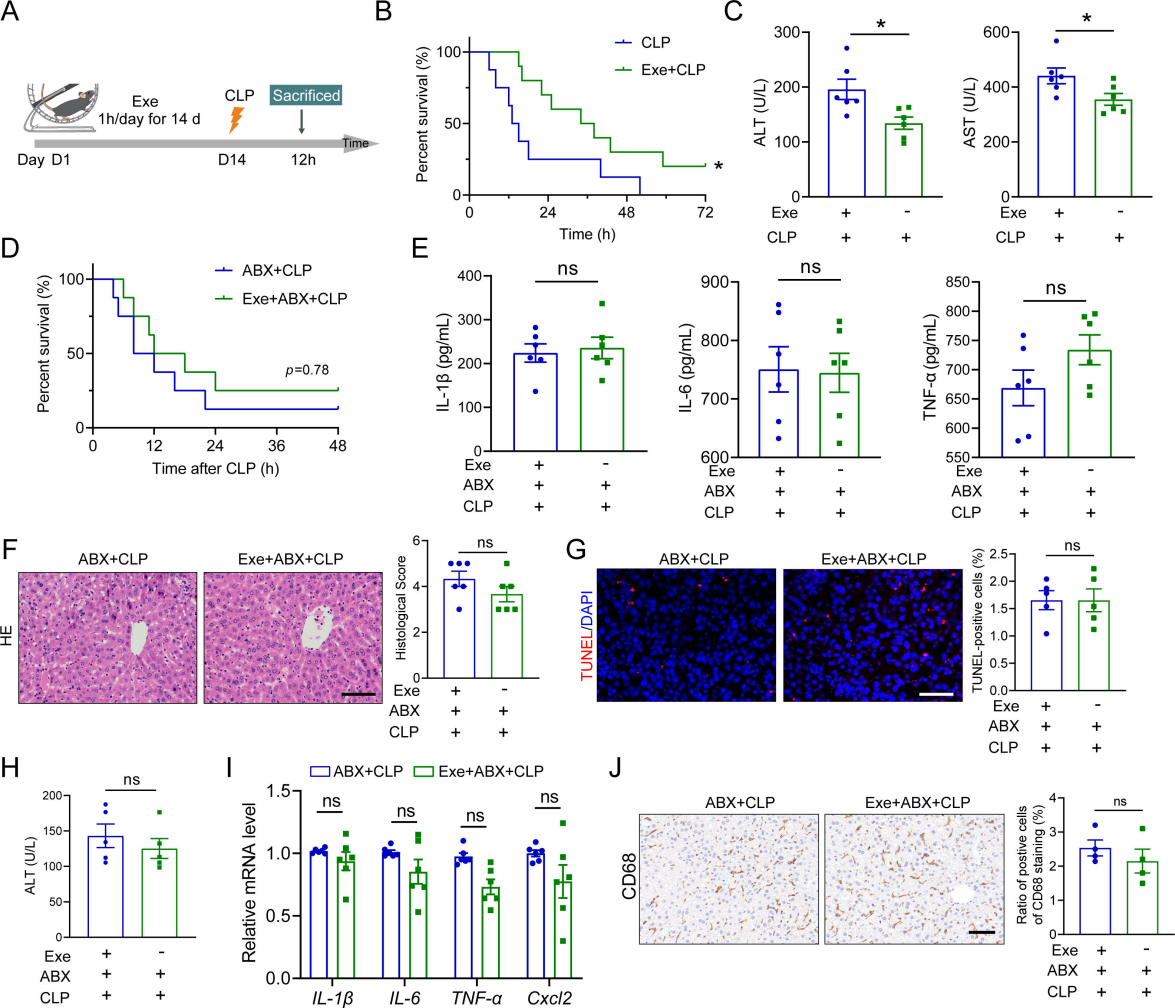
Gut microbiota plays a role in the protective effect of Exe against SALI. (**A**) Schematic diagram of the experimental approach: mice underwent a 1-h daily exercise training for 14 consecutive days before the CLP treatment. (**B**) The survival rates were determined using Kaplan-Meier curves in the mice pretreated with or without Exe, *n* = 12. (**C**) Plasma levels of ALT and AST in the mice pretreated with or without Exe, *n* = 6. (**D**) The survival rates were assessed by Kaplan-Meier curves, *n* = 12. (**E**) The levels of TNF-α, IL-1β, and IL-6 in plasma from antibiotic cocktail (ABX)-pretreated septic mice with or without Exe treatment, *n* = 6. (**F**) Representative H&E staining images and the histological score for lives in ABX-pretreated septic mice with or without Exe treatment, scale bar: 100 µm, *n* = 6. (**G**) TUNEL staining of the liver from ABX-pretreated septic mice with or without Exe treatment and quantification of dead cells, scale bar: 100 µm, *n* = 5. (**H**) Plasma levels of ALT from ABX-pretreated septic mice with or without Exe treatment, *n* = 6. (**I**) The mRNA levels of *IL-6*, *IL-1β*, *TNF-α*, and *Cxcl2* in the liver of ABX-pretreated septic mice with or without Exe treatment, *n* = 6. (**J**) Immunochemistry staining and quantification of CD68^+^ cells in the liver sections from ABX-pretreated septic mice with or without Exe treatment, scale bar: 100 µm, *n* = 4. All data are expressed as mean ± SEM. *P < 0.05, ^ns^*P* > 0.05, as indicated.

To investigate the potential of exercise training (Exe) in preventing SALI, mice were exposed to treadmill running for 2 weeks before CLP treatment (Fig. 1A). The implementation of exercise training had no impact on energy intake and body weight (Fig. S1A and B). We found that the septic Exe group showed a significantly longer survival rate compared to the septic control mice (Fig. 1B). Consistent with previous studies (13), our results demonstrated significant inhibition of liver damage in the septic Exe group compared to the septic control mice, as evidenced by reduced levels of serum transaminases (ALT and AST), histopathological alterations, and cell death (Fig. 1C, S2A-B). The participation of systemic inflammation emerges as a pivotal factor contributing to the outcome of sepsis (14). The impact of exercise training on the inflammatory response was further demonstrated through the observation of reduced recruitment of macrophages in the liver, as well as decreased hepatic mRNA (*IL-6*, *TNF-α*, *IL-1β*, and *Cxcl2*) and serum concentrations of inflammatory cytokines, including TNF-α, IL-1β, and IL-6 in CLP Exe mice (Fig. S2C through E). These results demonstrate that exercise training possesses the potential to ameliorate hepatic injury and systemic inflammation in septic mice.

To investigate the association between exercise training-induced protective effects against SALI and gut microbiota, we administered oral antibiotics to Exe-treated mice for 5 days before CLP to deplete the gut microbiota. Following ABX pretreatment, the survival curves of septic mice with or without exercise training exhibited no significant differences (Fig. 1D). We found that septic Exe mice with ABX treatment exhibited the loss of beneficial effects of exercise training in SALI mice, including ALT and AST (Fig. 1H; Fig. S3A), histopathologic changes (Fig. 1F), and cell death (Fig. 1G), suggesting that the beneficial effects of exercise training on SALI were compromised by ABX treatment. In agreement with this, no disparities were observed in serum concentrations and hepatic mRNA levels of inflammatory cytokines and hepatic recruitment of macrophages between septic Exe or Ctrl mice with ABX treatment (Fig. 1E, I and J). Hence, these findings substantiate that gut microbiota plays a vital role in the protective effects of exercise training against SALI.

### Exe-associated gut microbiota contributes to the beneficial effect of Exe on SALI

**Fig 2:**
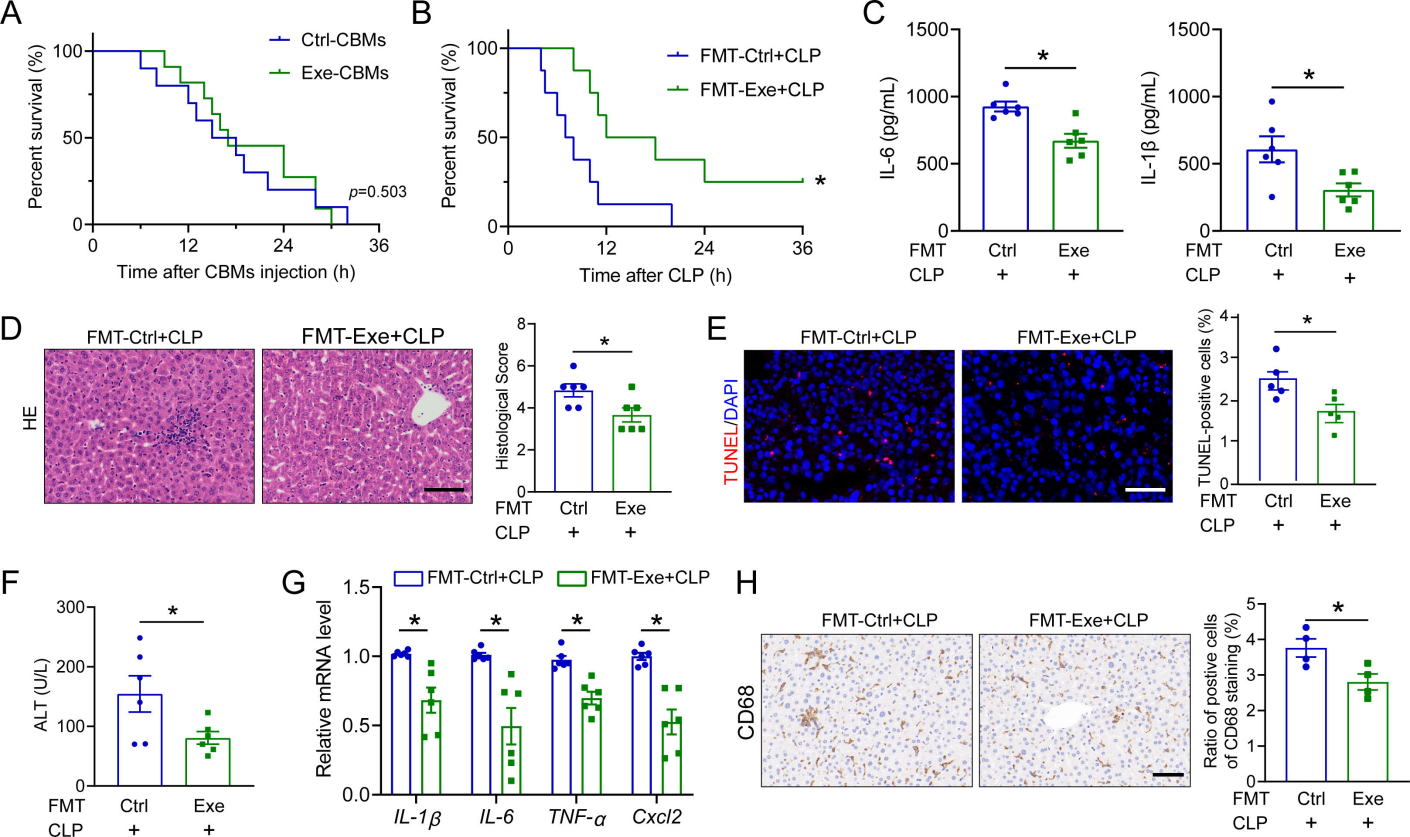
Fecal microbial transplantation from exercise training-treated mice protects against SALI. (**A**) The survival curve of septic mice was determined by intraperitoneal injections of CBMs derived from Exe and Ctrl mice. *n* = 12. (**B**) The survival curves of CLP-induced mice received a fecal suspension from Ctrl and Exe mice, *n* = 12. (**C**) The levels of TNF-α, IL-1β, and IL-6 in plasma from septic mice received fecal suspension from Ctrl and Exe mice, *n* = 6. (**D**) H&E staining and histological score for the livers in septic mice received fecal suspension from Ctrl and Exe mice, scale bar: 100 µm, *n* = 6. (**E**) TUNEL staining of septic mice received fecal suspension from Ctrl and Exe mice, scale bar: 100 µm, *n* = 5. (**F**) Plasma levels of ALT from septic mice received fecal suspension from Ctrl and Exe mice, *n* = 6. (**G**) The mRNA levels of *IL-6*, *IL-1β*, *TNF-α*, and *Cxcl2* in the liver of septic mice received fecal suspension from Ctrl and Exe mice, *n* = 6. (**H**) Immunochemistry staining and quantification of CD68^+^ cells in the liver sections from septic mice received fecal suspension from Ctrl and Exe mice, scale bar: 100 µm, *n* = 4. All data are expressed as mean ± SEM. *P < 0.05, as indicated.

To exclude the possibility that the altered microbiota as the intestinal source of infection may directly account for the protective effects of exercise training in SALI, we extracted cecal bacteria from Exe and Ctrl mice. Septic mice induced by separate intraperitoneal injections of cecal bacterial mixtures (CBMs) from Exe and Ctrl mice exhibited comparable susceptibility (Fig. 2A). Based on these observations, we hypothesized that gut microbiota contributed to the protective effects of exercise training against SALI. To validate our hypothesis, we conducted a fecal microbial transplantation experiment. The results of the survival analysis demonstrated that the group receiving feces from Exe donors exhibited enhanced resistance against sepsis in comparison to the group receiving feces from Ctrl donors (Fig. 2B). Furthermore, mice receiving fecal samples from the Exe-treated group showed reduced hepatic injury compared to those receiving samples from the control group after CLP treatment, as indicated by liver histopathology and serum levels of ALT and AST (2D, 2F, S3B). In line with these results, TUNEL staining showed that mice that received fecal samples from the Exe mice exhibited reduced cell death (Fig. 2E). Mice that received fecal samples from Exe donors exhibited reduced plasma concentrations of cytokines (Fig. 2C; Fig. S3C), hepatic inflammatory factors (Fig. 2G), and hepatic recruitment of macrophages (Fig. 2H) compared to recipients of fecal samples from Ctrl donors following CLP. Thus, these data indicate the protective effects of exercise training against SALI are at least partially dependent on gut microbiota.

### Exercise training enriches intestinal Ligilactobacillus and BA in mice

**Fig 3:**
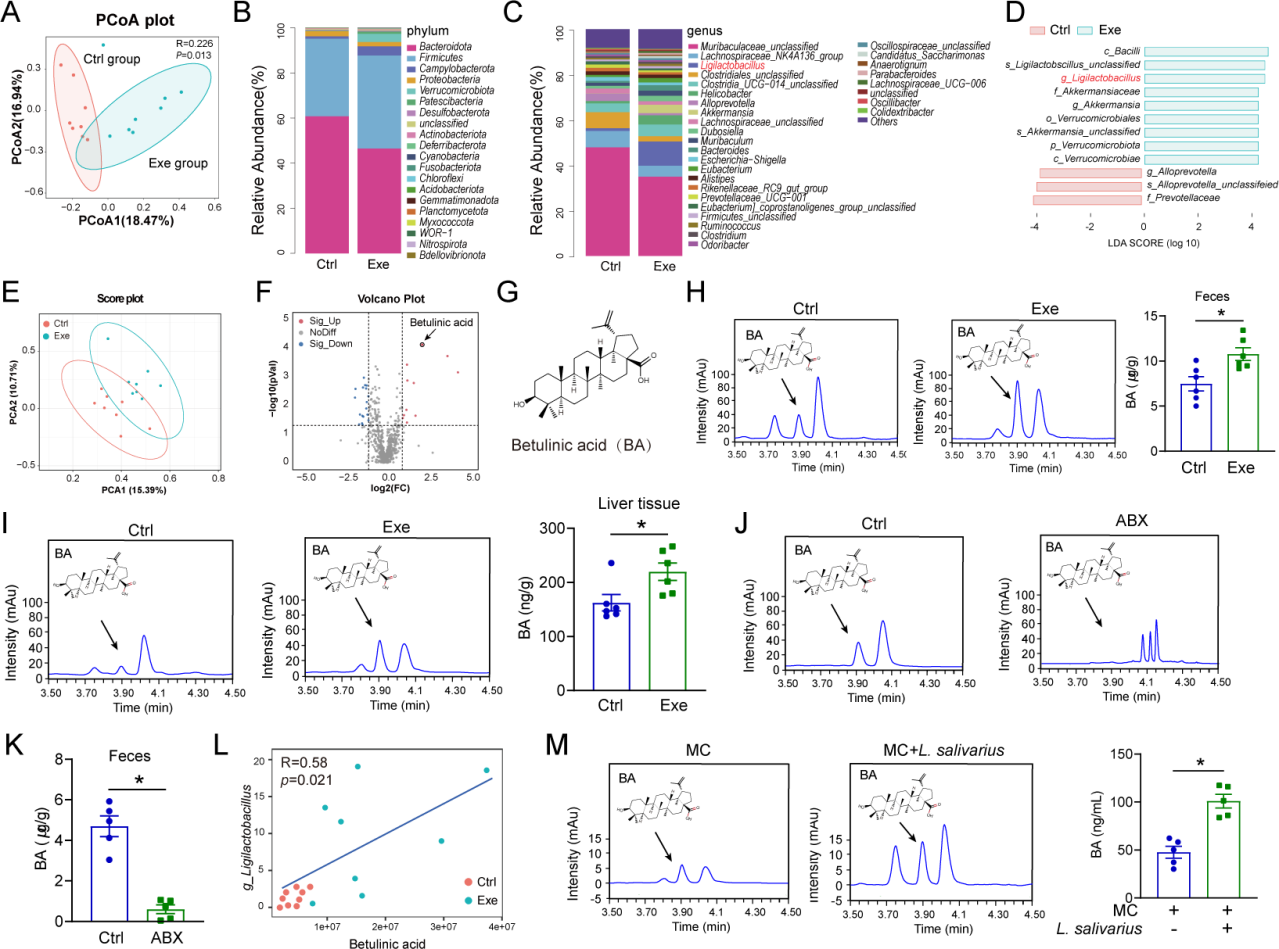
Metabolites and microbiota in the gut may work together to facilitate the preventive effect of Exe in SALI. (**A**) Principal component analysis (PCOA) of the gut microbiota in Ctrl and Exe-treated mice, *n* = 8. (**B**) Relative abundance of gut microbiota at the phylum level in Ctrl and Exe mice, *n* = 8. (**C**) Relative abundance of gut microbiota at the genus level in Ctrl and Exe mice, *n* = 8. (**D**) LDA along with effect size measurements in gut microbiota from Ctrl and Exe mice. (**E**) The PCA analysis of gut metabolites from Ctrl and Exe mice, *n* = 8. (**F**) The volcano plot of gut metabolites from Ctrl and Exe mice. (**G**) Molecular structure of BA. (**H**) BA concentration in feces from Ctrl and Exe mice, *n* = 6. (**I**) BA concentration in liver tissues from Ctrl and Exe mice, *n* = 6. (**J, K**) BA concentration in feces from mice with or without ABX treatment, *n* = 6. (**L**) Correlation between BA concentration and abundance of *Ligilactobacillus* in mouse feces, *n* = 8. (**M**) Medium BA concentration in the culture of *L. salivarius*. Mouse chow was mixed with PBS and incubated at 37°C for 24 h, either in the presence or absence of *L. salivarius*, *n* = 5. All data are expressed as mean ± SEM. *P < 0.05, as indicated.

To investigate the underlying mechanisms by which changes in microbiota induced by exercise training inhibit SALI in mice, we conducted a 16S rRNA gene sequencing analysis. We observed a distinct modification in the gut microbiota composition after CLP in both the Exe and Ctrl groups (Fig. 3A, S4B). At the phylum level, Exe increased the abundance of *Firmicutes* and *Campylobacterota* while decreasing the abundance of *Bacteroidota* and *Proteobacteria* presence (Fig. 3B). At the genus level, Exe significantly increased the relative abundance of *Ligilactobacillus* and *Akkermansia*, while simultaneously reducing the abundance of *Muribaculaceae* and *Lachnospiraceae_NK4A136* (Fig. 3C). Based on the Phylogenetic Investigation of Communities by Reconstruction of Unobserved States (PICRUSt) analysis, several microbial metabolic pathways in the Exe mice exhibited higher levels of enrichment (Fig. S1C). The linear discriminant analysis (LDA) revealed a significant increase in the abundance of *Ligilactobacillus* and *Akkermansia* in Exe mice, while *Alloprevotella* and *prevotellaceae* were found to be enriched in Ctrl mice (Fig. 3D).

Given the potential involvement of gut-derived metabolites in the protective effects of exercise training against SALI, we subsequently conducted a metabolomic analysis to examine how exercise training affects the metabolic profiles of gut microbiota. As expected, the principal component analysis (PCA) showed a significant difference in the gut microbiota metabolic profiles between the two groups (Fig. 3E). The volcano plot revealed significant alterations in 1,162 metabolites (*P* < 0.05) present in the feces of Exe mice as compared to Ctrl mice (Fig. 3F). The focus of our investigation was directed toward BA, a potent anti-inflammatory agent (Fig. 3G) because the abundance of BA was significantly elevated in Exe mice compared to Ctrl mice (Fig. S4D). The abundance of BA in the feces and liver tissues of Exe mice was further confirmed by LC-MS/MS. We found that BA was enriched in the feces and livers of the Exe mice compared to Ctrl mice (Fig. 3H and I). We next verify whether BA is a metabolite of intestinal microbiota; the intestinal BA was decreased by gut microbiota depletion with ABX treatment (Fig. 3J and K), suggesting that BA production was dependent on gut microbiota. To establish a correlation between these metabolites and the potential metabolic activities of gut microbes, integrative analyses of altered bacteria and metabolites were performed. The correlation between the abundances of *Ligilactobacillus* and BA in matched samples was further confirmed through integrated multiomics analysis (Fig. 3L), suggesting that *Ligilactobacillus* has the potential to stimulate BA generation. The *ex vivo* fermentation experiments were then conducted. The mouse chow was mashed and dissolved in PBS, followed by an anaerobic culture with live *L. salivarius* for 24 h. As expected, the concentration of BA in the culture supernatants of *L. salivarius* was found to be higher compared to that in the culture medium (Fig. 3M), indicating the ability of *L. salivarius* to induce BA production from mouse chow. Together, these data reveal that exercise training could enrich intestinal *Ligilactobacillus* and increase BA production, indicating that metabolites and microbiota in the gut may work together to facilitate the preventive effect of Exe on SALI.

### BA ameliorates SALI in a murine model

**Fig 4:**
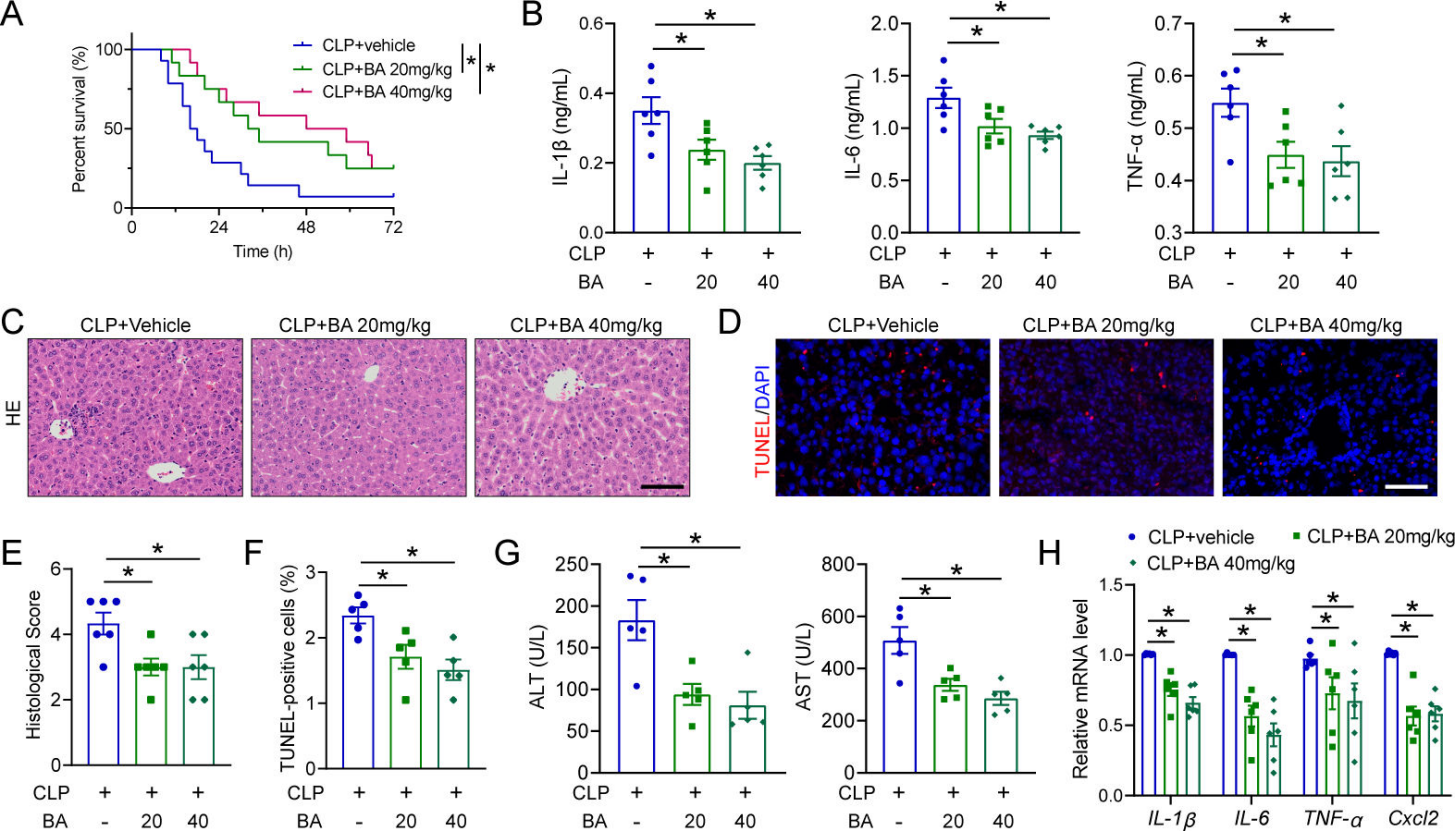
BA ameliorates SALI in a murine model. (**A**) The survival curves of septic mice pretreated with either 20 or 40 mg/kg BA, *n* = 12–14. (**B**) The levels of TNF-α, IL-1β, and IL-6 in plasma from septic mice with or without BA treatment, *n* = 6. (**C, E**) H&E staining and histological score for the liver in septic mice with or without BA treatment, scale bar:100 µm, *n* = 6. (**D, F**) TUNEL staining of septic mice with or without BA treatment, scale bar:100 µm, *n* = 5. (**G**) Plasma levels of ALT and AST from septic mice with or without BA treatment, *n* = 6. (**H**) The mRNA levels of *IL-6*, *TNF-α*, *IL-1β*, and *Cxcl2* in the liver of septic mice with or without BA treatment, *n* = 6. All data are expressed as mean ± SEM. *P < 0.05, as indicated.

BA is a naturally pentacyclic triterpenoid, possessing diverse biological and medicinal properties, including anti-cancer, anti-malarial, anti-oxidant, and anti-inflammatory activities (15). Next, we investigated the regulatory role of BA in SALI. BA treatment of 20 mg and 40 mg/kg did not induce any adverse effects in mice (Fig. S3D). Mice pretreated with BA exhibited significantly prolonged survival time compared to the CLP control mice (Fig. 4A). Moreover, our findings demonstrated that, compared to the CLP control mice, BA treatment significantly attenuated liver damage, as evidenced by reduced levels of serum ALT and AST (Fig. 4G), amelioration of histopathologic alterations (Fig. 4C and E), and suppression of cell death (Fig. 4D and F). Furthermore, we investigated whether BA treatment could attenuate the septic inflammation. We found that the secretion of proinflammatory cytokines IL-6, IL-1β, and TNF-α in mouse serum was significantly reduced following BA treatment (Fig. 4B). In line with these findings, the mRNA levels of inflammation markers and hepatic macrophage recruitment were significantly attenuated in BA-treated mice compared to CLP control mice (Fig. 4H; Fig. S4A). We, therefore, conclude that BA treatment mitigates the liver injury and systemic inflammation in septic mice.

### BA suppresses the NLRP3 inflammasome activation in sepsis

**Fig 5:**
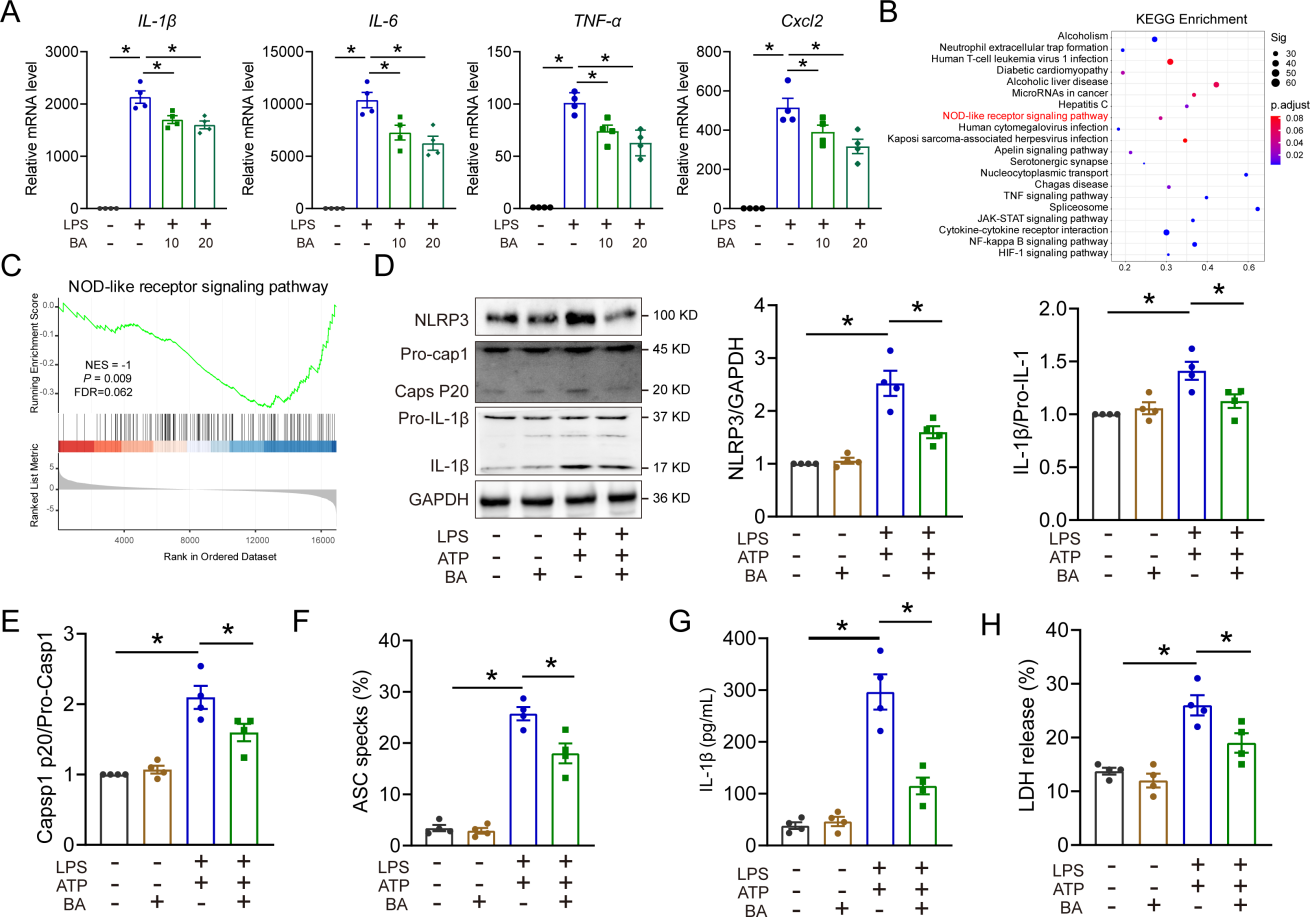
BA suppresses NLRP3 inflammasome activation in sepsis. (**A**) The mRNA levels of *IL-6*, *IL-1β*, *TNF-α*, and *Cxcl2* in BMDMs after LPS stimulation for 3 h with or without BA (10 or 20 µM), *n* = 4. (**B**) Significantly changed pathways of the differentially expressed upregulated genes based on the KEGG signaling pathway analysis in BMDMs after LPS stimulation for 3 h with or without BA (20 µM), *n* = 3. (**C**) GSEA is significantly expressed in the NOD-like receptor signaling pathway. (**D, E**) Expression of NLRP3 inflammasome-associated proteins NLRP3, Caps P20, and IL-1β in BMDMs by Western blot. After priming with LPS and BA as mentioned above, BMDMs were stimulated with 5 mM ATP for 30 min, *n* = 4. (**F**) Quantification of ASC oligomerization in BMDMs, *n* = 4. (**G**) Inhibitive effect of BA on IL-1β release from BMDMs, *n* = 4. (**H**) Inhibitive effect of BA on LDH release from BMDMs, *n* = 4. All data are expressed as mean ± SEM. *P < 0.05, as indicated.

Macrophage activation plays a crucial role in the initiation of cytokine storms during the early stage of sepsis (16). Therefore, we investigated the impact of BA on LPS-induced macrophage activation. Our results revealed that BA treatment at 10 and 20 µM dose effectively suppressed the expression of proinflammatory cytokines in LPS-stimulated BMDMs (Fig. S4E). The *in vitro* experiments demonstrated that treatment with BA effectively suppressed the expression of proinflammatory cytokines in LPS-stimulated BMDMs (Fig. 5A). To elucidate the underlying mechanisms by which BA mitigates inflammation in LPS-treated macrophages, we conducted the transcriptomic analysis. Our findings revealed 1,302 upregulated and 968 downregulated genes in BA-treated BMDMs compared to control BMDMs induced by LPS. The Kyoto Encyclopedia of Genes and Genomes (KEGG) enrichment analysis revealed that BA exerts inhibitory effects on the NOD-like receptor signaling pathway, tumor necrosis factor (TNF) signaling, cytokine-cytokine receptor interaction, and NF-Kappa B signaling pathways (Fig. 5B). Gene Set Enrichment Analysis (GSEA) analysis showed that NOD-like receptor signaling pathway was modulated by BA in BMDMs treated with LPS (Fig. 5C). In support of this, BA administration effectively suppressed the activation of NLRP3 inflammasome induced by LPS plus ATP, as evidenced by reduced expression levels of NLRP3, caspase-1 p20, and IL-1β (Fig. 5D and E). Moreover, the Apoptosis-associated speck-like protein containing a CARD (ASC) oligomerization induced by LPS plus ATP was suppressed after BA administration in BMDMs (Fig. 5F; Fig. S4F). The administration of BA effectively attenuated the release of LDH and IL-1β induced by LPS plus ATP (Fig. 5G and H), indicating its potential as a suppressor of NLRP3 inflammasome activation in septic macrophages. Moreover, BA treatment effectively suppressed the activation of NLRP3 inflammasome in the liver of septic mice (Fig. S4G). Collectively, these results indicate that BA suppresses the NLRP3 inflammasome activation in sepsis.

### BA inhibits the NLRP3 inflammasome activation in macrophages by directly targeting hnRNPA2B1

**Fig 6:**
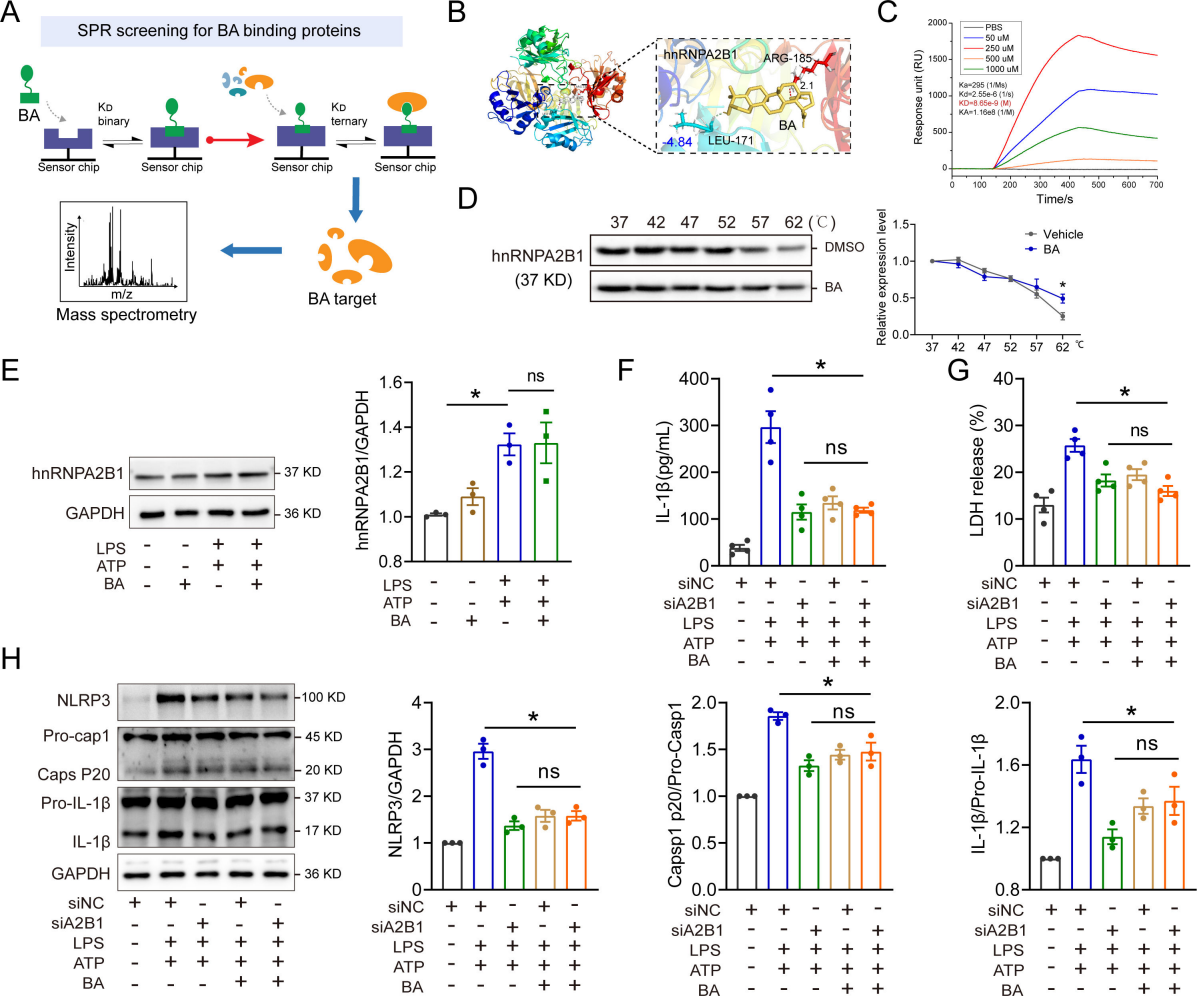
BA inhibits NLRP3 inflammasome activation in macrophages by directly targeting hnRNPA2B1. (**A**) Schematic diagram of SPR screening for BA binding proteins. (**B**) Molecular docking analysis of BA with hnRNPA2B1 (PDB:7WM3). (**C**) SPR analysis for hnRNPA2B1 protein with different doses of BA. (**D**) Western blot analysis of hnRNPA2B1 degradation in BMDMs lysates treated with or without 20 µM BA, *n* = 3. (**E**) Expression of hnRNPA2B1 in BMDMs by Western blot. After being primed with LPS and BA as described above, BMDMs were stimulated with 5 mM ATP for 30 min *n* = 3. (**F**) Inhibitive effect of BA on IL-1β release from BMDMs, *n* = 4. (**G**) Inhibitive effect of BA on LDH release from BMDMs, *n* = 4. (**H**) Expression of NLRP3 inflammasome-associated proteins NLRP3, Caps P20, and IL-1β in BMDMs by western blot. BMDMs were subjected to a 36 h transfection with siRNA specifically targeting hnRNPA2B1 (siA2B1) or control siRNA (siNC). Following this, BMDMs were treated as described above, *n* = 3. All data are expressed as mean ± SEM. *P < 0.05, ^ns^ *P* > 0.05, as indicated.

To further investigate the underlying molecular mechanisms by which BA modulates the activation of NLRP3 inflammasome, we employed the SPR-LC-MS/MS analysis (Fig. 6A). The mass spectrum analysis revealed that heterogeneous nuclear ribonucleoprotein A2B1 (hnRNPA2B1) was identified as a potential target of BA, which has been well linked to the inflammatory response (17). Molecular docking analysis suggested a potential direct interaction between BA and the protein hnRNPA2B1 (Fig. 6B). The BA-hnRNPA2B1 interactions were further validated through the implementation of SPR interaction and affinity analysis. The SPR interaction and affinity analysis revealed a significant positive direct interaction between BA and hnRNPA2B1 in a dose-dependent manner (Fig. 6C). In addition, a cellular thermal shift assay (CETSA) was conducted to assess the interactions between BA and hnRNPA2B1. As expected, the administration of BA effectively attenuated the degradation rate of hnRNPA2B1 in heat-denatured BMDMs when compared to the vehicle-treated group (Fig. 6D). These findings elucidate the direct interaction between BA and hnRNPA2B1 in macrophages.

hnRNPA2B1, a member of the hnRNP family of RNA binding proteins, exerts its influence on various biochemical functions of RNA, including localization, shearing, stability, and mRNA trafficking (18, 19). Our results found that BA did not regulate the protein and mRNA expression level of hnRNPA2B1 (Fig. 6E; Fig. S5A). Moreover, BA could potentially induce nuclear accumulation of specific pri-miRNAs by inhibiting the activity of hnRNPA2B1 (Fig. S5B). These data suggest that BA serves as a potent hnRNPA2B1 inhibitor. A recent study demonstrated that the significant involvement of hnRNPA2B1 in the innate immune responses against DNA viral infections (20). To validate the hnRNPA2B1-dependent effects of BA on NLRP3 inflammasome activation, we employed RNA interference to silence hnRNPA2B1 in BMDMs. Compared to the LPS plus ATP group, the release of LDH and IL-1β was reduced upon hnRNPA2B1 knockdown in BMDMs subjected to LPS plus ATP treatment (Fig. 6F and G). In addition, the knockdown of hnRNPA2B1 resulted in decreased expression levels of NLRP3, caspase-1 p20, and IL-1β in BMDMs treated with LPS plus ATP (Fig. 6H). Knockdown of hnRNPA2B1 also concurrently attenuated ASC oligomerization in BMDMs treated with LPS plus ATP (Fig. S5D). Although the activation of NLRP3 inflammasome in LPS plus ATP-induced BMDMs was reduced by BA or hnRNPA2B1 knockdown, the additional beneficial effect of BA on the NLRP3 inflammasome activation was not observed when hnRNPA2B1 siRNA was used (Fig. 6F through H). Moreover, BA treatment significantly reduced the mRNA expression of *Nlrp3* and *IL-1β* while having no effect on *Caspase1* in BMDMs treated with LPS plus ATP. Compared to the LPS plus ATP group, the mRNA expression of *Nlrp3* was reduced upon hnRNPA2B1 knockdown in BMDMs subjected to LPS plus ATP treatment (Fig. S5E). Collectively, these results indicate that the protective effect of BA against sepsis is, at least partially, mediated through its targeting of hnRNPA2B1.

## DISCUSSION

**Fig 7:**
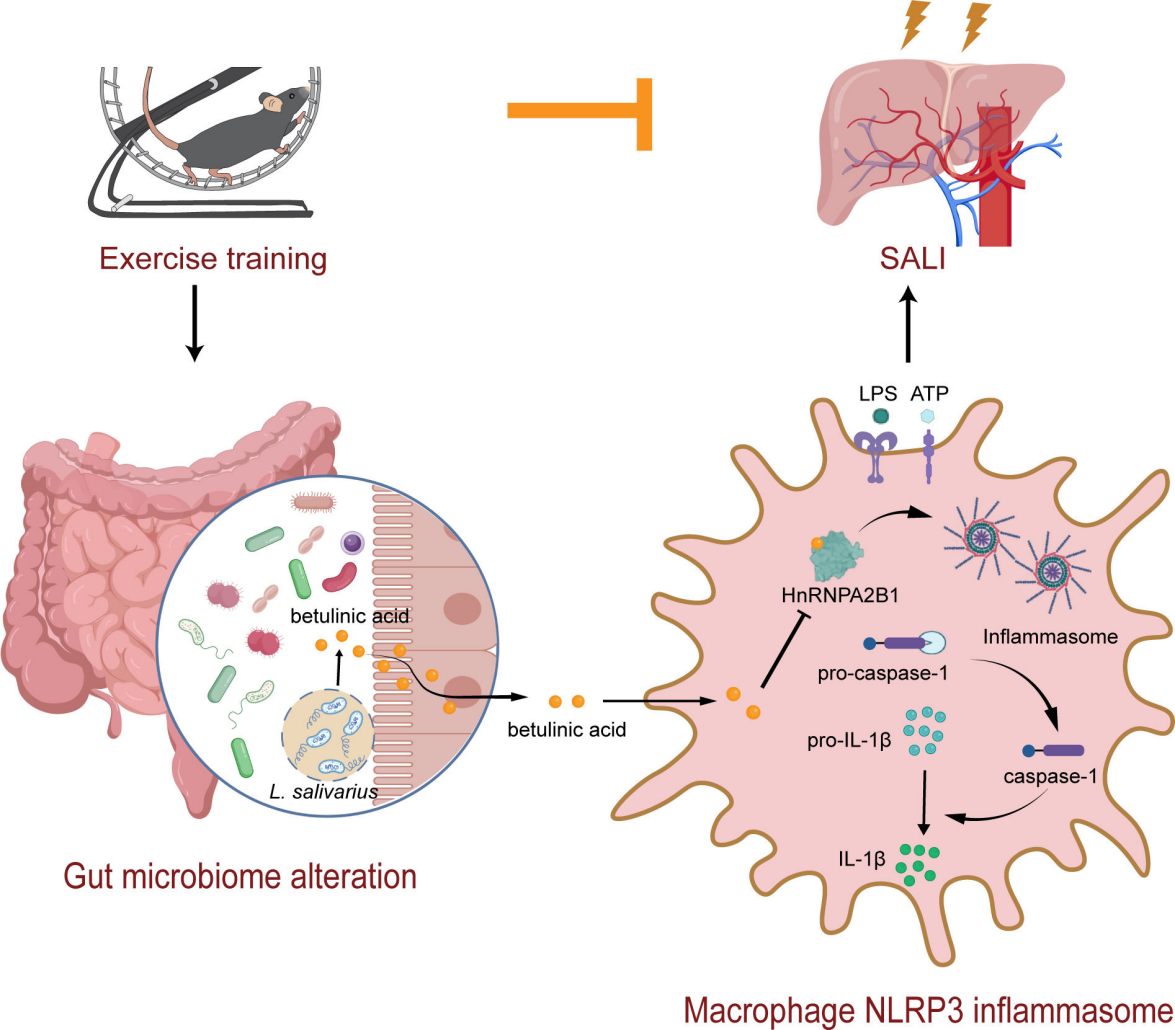
Working model: gut microbe-derived betulinic acid (BA) mediated the protective effects of exercise training on sepsis-induced acute liver injury. Exercise training increased the abundance of *Ligilactobacillus* and its metabolite BA. A mechanistic study revealed that BA inhibited NLRP3 inflammasome activation in macrophages by directly binding to hnRNPA2B1.

This study has made significant contributions to our comprehension of the underlying mechanisms through which exercise training protects against SALI in mice. The positive impact of exercise training on the gut microbiome has gained widespread recognition (21, 22). Here, we revealed that exercise training exerted a substantial anti-inflammatory impact by modulating the gut microbiome in SALI mice. Moreover, the alterations in gut microbiota induced by preoperative exercise training conferred a protective effect on SALI and resulted in elevated concentrations of the microbial metabolite BA. BA treatment effectively alleviated SALI both *in vivo* and *in vitro* by inhibiting NLRP3 inflammasome activation in macrophages by direct targeting of hnRNPA2B1 (Fig. 7). These findings propose an innovative strategy for mitigating SALI through modulation of the gut microbiome, offering a potential therapeutic target for sepsis management.

Engaging in multiple sessions of moderate physical activity has been proven to enhance immune system monitoring and provide numerous advantages for human health (23, 24). However, the potential mechanisms underlying the potential counteraction of SALI by exercise training remain poorly elucidated. Here, 16S rRNA gene sequencing revealed a distinct separation between gut microbiota in the Exe and Ctrl groups. Notably, probiotics *Ligilactobacillus* and *Akkermansia* were enriched by Exe treatment. These results indicated that Exe-induced gut microbiota alteration might contribute to its preventive effect on SALI. Through intraperitoneal injections of CBMs from Exe and Ctrl mice, we initially ruled out the possibility that alterations in gut microbiota induced by exercise training could affect susceptibility to CLP-induced sepsis. After pretreatment with ABX, we observed a comparable mortality rate between Exe mice and Ctrl septic mice. Furthermore, the fecal microbiota transplantation (FMT) experiments validate that the transplantation of fecal microbiota from Exe donors effectively reduces mortality risk in septic mice compared to that from Ctrl mice. Thus, these data indicate the microbiota play a significant role in mediating the protective effects of exercise training against SALI.

Given the pivotal role of gut microbiota-derived metabolites in the regulation of health and disease (24, 25), we subsequently performed a non-targeted metabolite identification analysis on fecal samples obtained from Exe-treated mice and Ctrl mice. We found that exercise training enriched intestinal BA in mice. Through comprehensive multi-omics analysis, we have further substantiated the ability of *L. salivarius* to induce BA liberation from mouse chow. Our findings demonstrate that exercise training promotes the enrichment of intestinal *Ligilactobacillus* and enhances BA production, but the mechanistic mechanisms underlying the generation of BA by *L. salivarius* remain unidentified. BA, a lupine-type pentacyclic triterpene, has been extensively documented in traditional Chinese herbal medicine (26). A previous study suggests that BA can protect against Carr-induced paw edema by reducing inflammation and oxidative stress (27). Another study indicates that the oral administration of BA exhibits the potential to ameliorate colon inflammation and fibrosis in inflammatory bowel disease (28). Our data demonstrate that BA effectively inhibits the activation of NLRP3 inflammasome in macrophages by targeting hnRNPA2B1. These results further enhance our understanding of the function of *L. salivarius* in sepsis. However, we could not exclude the possibility that other crucial metabolites derived from *L. salivarius* may also confer protection against SALI. Extensive research is imperative to assess whether the other essential metabolites derived from *L. salivarius* could synergistically contribute to the treatment of sepsis.

The NLRP3 inflammasome serves as a cytosolic multiprotein caspase-activating complex platform involved in innate immunity, essential for the maturation and release of interleukin (IL)-1β and IL-18 (29, 30). Accumulating data suggest that the NLRP3 inflammasome plays a key role in the pathogenesis of sepsis (31). It has been reported that NLRP3 inflammasome and its associated signaling pathways may exert regulatory effects on inflammation, autophagy, apoptosis, and pyroptosis in sepsis-induced myocardial dysfunction (32). Another study demonstrates that inhibition of the NLRP3/IL-1β pathway mitigates cardiac atrophy and cardiomyopathy in septic mice (33). Here, we found that hnRNPA2B1 positively regulated NLRP3 inflammasome activation in macrophages, suggesting that targeting its activity could offer potential therapeutic strategies for sepsis. The RNA-binding proteins hnRNPA2B1 enhance disease susceptibility by regulating the splicing and stability of target mRNA (34, 35). The SPR-LC-MS/MS analysis revealed that hnRNPA2B1 was identified as a potential BA target. The binding of BA with hnRNPA2B1 was observed in a dose-dependent manner through SPR and CETSA experimentation. As a regulator of miRNA precursors, hnRNPA2B1 plays a crucial role in overseeing the conversion process from pri-miRNAs to pre-miRNAs within the nucleus (36). Here, we found that BA treatment could lead to nuclear accumulation of specific pri-miRNAs through inhibition of hnRNPA2B1 activity. Our findings support that BA is a natural antagonist of hnRNPA2B1, inhibiting NLRP3 inflammasome activation by suppressing hnRNPA2B1 activity. These findings suggest that BA, an inhibitor specifically targeting hnRNPA2B1, holds promise as a potential therapeutic agent for the treatment of SALI.

In summary, exercise training exerts a protective effect against SALI by modulating the composition of gut microbiota and enhancing the secretion of defensive metabolites. Exercise training is able to increase the abundance of *Ligilactobacillus* and its metabolite BA. Functionally, BA treatment inhibits NLRP3 inflammasome activation in macrophages by directly binding to hnRNPA2B1, thereby providing the ultimate protection against SALI. Our findings underscore the protective effect of exercise training against sepsis and highlight the potential therapeutic role of BA in combating lethal sepsis.

## MATERIALS AND METHODS

### Animal models

Male C57BL/6J mice, aged 6–8 weeks, were housed under conditions of *ad libitum* access to food and water. The induction of polymicrobial sepsis was achieved using the cecal ligation and puncture (CLP) method, as described previously (37). In brief, the mice were initially administered anesthesia before CLP treatment by ligating and puncturing their cecum twice using an 18 G needle. The survival rate was observed every hour during the first 48 h period. For exercise training, mice were randomly assigned to two activity groups: exercise training (Exe) or sedentary control (Ctrl). The Exe mice underwent a treadmill exercise routine as described previously (38), starting at 5 m per minute for 10 min and then increasing to 15 m per minute for 60 min once daily for 14 days. In contrast, the Ctrl mice were housed in cages without any physical activity. For the administration of BA (HY-10529, MedChemExpress), mice were orally given a dosage of 20 or 40 mg/kg BA 2 h prior to performing the CLP surgery. For the treatment with an antibiotic cocktail (ABX), mice were orally administered vancomycin (100 mg/kg), neomycin sulfate (200 mg/kg), metronidazole (200 mg/kg), and ampicillin (200 mg/kg) (Macklin, Shanghai, China) once daily for 5 days.

### Cell and bacterial culture

Bone marrow-derived macrophages (BMDMs) were isolated and differentiated into macrophages as previously described (39). In brief, BMDMs were generated by culturing bone marrow cells obtained from male mice in Dulbecco’s modified Eagle’s medium (DMEM) supplemented with 1% penicillin–streptomycin (15140-122, Gibco) and 20 ng/mL macrophage colony-stimulating factor (M-CSF, 416 mL, R&D Systems). *L. salivarius* was cultured in Man Rogosa Sharpe (MRS) medium (HKM, China) at 37°C under anaerobic conditions.

### Fecal microbiota transplantation

The FMT experience was performed as previously described (40). Briefly, fecal samples were obtained after exercise training for 14 days. The gut microbiota in mice was eradicated using an antibiotics cocktail administered orally for 5 days. Subsequently, fecal pellets were suspended and homogenized in PBS at a density of 0.125 g/mL, followed by daily administration of the fecal supernatant to mice via oral gavage for 3 days. Afterward, the mice were subjected to CLP.

### Mouse model of cecal bacterial mixture-induced septic peritonitis

The cecal contents were obtained from Exe or Ctrl mice as previously described (41), followed by resuspension in PBS at a weight-to-volume ratio of 1:10. The CBMs were pelletized through gradient centrifugation and then resuspended in PBS at a weight/volume ratio of 1:5 based on the initial weight of cecal contents. Finally, a solution containing 300 mL of CBMs suspension was intraperitoneally injected into mice.

### Fecal DNA extraction and 16S rRNA sequencing

The fecal contents from mice were collected and sent to LC-Bio-Hangzhou (China) for metagenomic analysis. The genomic DNA was extracted from feces, quantified, and prepared DNA libraries. To analyze microbial diversity, the universal amplicon primers (forward primer: 50-GTGTGYCAGCMGCCGCGGTAA-30; reverse primer: 50-CCGGACTACNVGGGTWTCTAAT-30) targeting the V4 region of bacterial 16S rRNA gene were employed. Then, the products underwent analysis by the Illumina high-throughput sequencing and NovaSeq 6000 platforms. The data extraction, splicing, and bioinformatics analysis were performed using QIIME2. Additionally, the raw sequences underwent quality control using QIIME2, an open-source project primarily developed in the Knight and Caporaso labs. The next step involved demultiplexing the sequences and clustering them into operational taxonomic units at the species level, with a 97% similarity threshold. Subsequently, QIIME2 was utilized to perform principal component analysis, alpha diversity analysis, and beta diversity analysis.

### Measurement of cytokines and serum biochemistry

The levels of alanine transaminase (ALT) and aspartate transaminase (AST) were measured using commercially available kits in accordance with the instructions provided by the manufacturers (JianCheng, Nanjing, China). The serum levels of IL-1β, TNF-α, and IL-6 were assessed utilizing commercially accessible kits (NeoBioscience, Beijing, China).

### Histopathological analysis and immunohistochemistry

For hematoxylin and eosin (H&E) staining, the liver tissue was preserved in a 4% paraformaldehyde solution, subsequently encased in paraffin, and finally cut into sections with a thickness of 5 µm. Tissue samples were subjected to H&E staining and analyzed under a light microscope (Olympus, Tokyo). To determine cell death, a commercially available kit (Beyotime Biotechnology, China) was utilized for conducting terminal deoxynucleotidyl transferase dUTP nick end labeling (TUNEL) staining. A solution containing 10 mM sodium citrate, 0.5% Tween 20, and adjusted to pH 6.0 was employed for immunohistochemical (IHC) staining to facilitate epitope retrieval. Each slide underwent incubation with anti-rabbit CD68 (Servicebio, Wuhan, China), followed by biotin-conjugated goat anti-rabbit IgG (Gene Tech, Shanghai, China). Diaminobenzidine tetrahydrochloride (Gene Tech, Shanghai, China) was utilized to visualize the immunoreactivity of each slide. All slides were examined and documented using a microscope (Leica DMi8, Wetzlar, Germany). For each slide, a minimum of six fields were randomly chosen for evaluation through ImageJ software.

### Metabolomics analysis

All samples were subjected to extraction using a mixture of methanol and water (4:1, vol/vol) with the addition of 0.02 mg/mL of 2-chloro-L-phenylalanine. The homogenization process was carried out for 6 min at a temperature of −10°C. Subsequently, the solution underwent ultrasonic extraction on ice for 30 min, followed by centrifugation at 15,000 × *g* for 15 min at a temperature of 4°C. For LC-MS/MS analysis, a volume of 20 µL from the resulting supernatant was utilized. The UHPLC-Q Exactive system (Thermo Scientific, USA) was utilized for LC-MS/MS analysis. The LC conditions included the use of an Acquity UPLC HSS T3 column (100 mm × 2.1 mm i.d., 1.8 µm; Waters, Milford). Solvent A consisted of a mixture of water and acetonitrile in a ratio of 95:5 [0.1% (vol/vol) formic acid], while solvent B comprised acetonitrile, isopropanol, and water in a ratio of 47.5:47.5:5 [0.1% (vol/vol) formic acid]. The centroid data were gathered within the range of 70–1,050 *m*/*z*, utilizing a resolution of 70,000. Progenesis QI (Waters Corporation, Milford, USA) was employed for peak selection and alignment to identify metabolic biomarkers exhibiting significant variations between the medium and supernatant groups.

### Western blotting analysis

The Pierce BCA protein assay kit (23225, Thermo) was utilized to determine the concentrations of proteins. Subsequently, 10% or 12% SDS-PAGE was employed to separate the protein samples, which were then transferred onto polyvinylidene fluoride (PVDF) membranes (IPVH00010, Millipore). The following antibodies were used: NLRP3 monoclonal antibody (15101, Cell Signaling Technology, USA), hnRNPA2B1 antibody (sc-53620, Santa Cruz Biotechnology, USA), GSDMD polyclonal antibody (YT7991, Immunoway, China), Caspase1 polyclonal antibody (YT5743, Immunoway, China), IL-1β polyclonal antibody (YT5201, Immunoway, China；63124, CST), and mouse GAPDH antibody (AC002, ABclonal, China). After washing with TBST solution, the membranes were incubated at 37°C for 1 h with secondary antibodies as follows: horse-anti-mouse IgG-HRP (7076, CST) and goat-anti-rabbit IgG-HRP (7074, CST). Finally, the ChemiDoc MP imaging system (Bio-Rad USA) with Image Lab 5.2.1 software was used to analyze the bands.

### Transient transfection

The siRNA and NC oligonucleotide sequences were obtained from GeneChem (Shanghai, China). The transfection of hnRNPA2B1 siRNA or control siRNA was carried out using the Lipofectamine RNAiMAX Transfection Reagent (13778150, Invigentech). The sequences targeted by siRNA were as stated below: control siRNA, sense 5′-UUCUCCGAACGUGUCACGUTT-3′ and antisense 5′-ACGU GACACGUUCGGAGAATT-3′; hnRNPA2B1 siRNA, sense 5′- GCUGUUUGUUGGUGGAAUUTT-3′ and antisense 5′- AAUUCCACCAACAAACAGCTT-3′. Cell samples were collected for analysis 48 h post-transfection.

### Quantitative real-time RT-PCR

Relevant RNA was isolated from liver tissue or cells utilizing TRIzol reagent using a one-step cDNA kit (Toyobo, Osaka, Japan). Relevant pri-miRNA were quantified by a TaqMan Pri-miRNA assay. The SYBR Green Realtime PCR Master Mix (manufactured by Toyobo, Osaka, Japan) was utilized for RT-PCR analysis in accordance with the provided instructions. The expression levels of the target genes were standardized using 18S RNA as a reference gene. Table S1 displays the primers utilized for each gene.

### SPR-LC-MS/MS assay

The surface plasmon resonance with liquid chromatography-tandem mass spectrometry (SPR-LC-MS/MS) technology was employed to identify the potential binding proteins of BA in BMDMs following a previously described method (42). In the BA group, we immobilized BA on a 3D Dextran chip and then allowed BMDMs lysates to flow as the liquid phase. Similarly, in the vehicle group, an equivalent amount of vehicles was immobilized on the chip with BMDMs lysates flowing as the liquid phase. To prepare negative control samples (NC), we incubated BMDMs lysates with 5 µM BA for 2 h at 4°C and subsequently fully inactivated them by incubating them for 1 h at 60℃. The NC samples were then used with BA immobilized on the 3D Dextran chip during surface plasmon resonance (SPR) analysis. Afterward, nonspecifically attached proteins on the chip surface were removed through washing before analyzing the enriched binding proteins using high-performance liquid chromatography-tandem mass spectrometry (HPLC-MS/MS). The resulting MS data were analyzed utilizing MaxQuant software (COX LAB, version 1.3.0.5), where peptides were identified through database searching and BLASTP analysis.

### Cellular thermal shift assay

In brief, BMDMs were collected and subjected to freeze-thaw lysis using liquid nitrogen after being pelleted and washed with PBS. The resulting lysate was divided equally into two tubes, one incubated with BA (20 µM) and the other with an equivalent volume of DMSO at room temperature for 2 h. Subsequently, both lysates were subjected to heat treatment at temperatures ranging from 37°C to 62°C for 3 min, followed by immediate cooling at room temperature for another 3 min. After centrifugation at 15,000 rpm for 15 min at 4°C, the supernatant was mixed with SDS-PAGE loading buffer and boiled before being analyzed through immunoblotting.

### Surface plasmon resonance

The quantitative assessment of the interaction between BA and hnRNPA2B1 was conducted using the PlexArray HT System (PlexArray, USA) that incorporates a 3D MES/HEMA sensor chip. The purified recombinant hnRNPA2B1 protein was immobilized using a protein chip, followed by activation with 10 mM NiSO4. Different levels of BA (0, 50, 250, 500, 1,000 µmol/L) were used as analytes. The data analysis was conducted using dedicated software provided by Plexera Bioscience for PlexArray HT.

### Molecular docking analysis

The crystal structure of the hnRNPA2B1 protein was retrieved from the Protein Data Bank (PDB ID 7WM3), while the structure of BA was acquired from PubChem (CID: 64971). The analysis of the binding sites between BA and hnRNPA2B1 was conducted using Schrodinger-Maestro software (version 11.1).

### Fluorescence staining

BMDMs were placed onto a petri dish and subsequently treated with 4% paraformaldehyde for 15 min at ambient temperature. After performing three PBS washes, a permeabilization procedure and block process were conducted. Subsequently, cells were subjected to overnight incubation at 4°C with an anti-ASC antibody (67824, Cell Signaling Technology, USA). The fluorescence intensity of ASC was measured by analyzing a random selection of six to ten fields using Image J.

### Transcriptome analysis

The TRIzol reagent was employed to extract the RNA from BMDMs. The total RNA samples were extracted using the TRIzol method, enabling both quantitative and quality evaluation. The Illumina Novaseq 6000 platform (Novogene Co., Ltd., Beijing, China) was utilized for library preparation, establishment, and sequencing. Then, gene expression levels were determined by aligning with the mm39 reference genome and subsequently converted into trusted platform module values for further analysis.

### Statistical analysis

Prism 9.5.1 (GraphPad Software) was utilized to conduct statistical analysis. Log-rank (Mantel-Cox) tests were employed to analyze survival studies. Statistical significance was assessed using a two-tailed Student’s *t*-test or one-way analysis of variance, followed by the Bonferroni post hoc test. The establishment of relevance significance was achieved by employing the technique of linear regression analysis. The reported values are presented as the mean ± SEM. The statistical significance was determined by considering *P* values < 0.05.

## Data Availability

The 16S rRNA sequencing data and transcriptome data are available in the SRA database under Bioproject numbers PRJNA1189502 and PRJNA1189842. All of the metabolomics data were deposited in the NGDC database under accession number PRJCA035256. Other data are available from the corresponding author upon reasonable request.

## SUPPLEMENTAL MATERIAL

Supplemental material mbio.03020-24-s0001.docx Table S1 and Fig. S1-S5.

ASM does not own the copyrights to Supplemental Material that may be linked to, or accessed through, an article. The authors have granted ASM a non-exclusive, world-wide license to publish the Supplemental Material files. Please contact the corresponding author directly for reuse.
