# Erratum for Tang et al., “Gut microbe-derived betulinic acid alleviates sepsis-induced acute liver injury by inhibiting macrophage NLRP3 inflammasome in mice”

**DOI:** 10.1128/mbio.02792-25

**Published:** 2025-09-18

**Authors:** Xuheng Tang, Tairan Zeng, Wenyan Deng, Wanning Zhao, Yanan Liu, Qiaobing Huang, Yiyu Deng, Weidang Xie, Wei Huang

## ERRATUM

Volume 16, no. 3, e03020-24, 2025, https://doi.org/10.1128/mbio.03020-24. Page 15, Author Affiliations, affiliation 1: “Guangdong Provincial People’s Hospital, Guangdong Academy of Medical Sciences” should read “Guangdong Provincial People’s Hospital (Guangdong Academy of Medical Sciences), Southern Medical University.”

