# Supplementary material for: Gut microbe-derived betulinic acid alleviates sepsis-induced acute liver injury by inhibiting macrophage NLRP3 inflammasome in mice
Source: mBio. 2025 Jan 31;16(3):e03020-24. doi: 10.1128/mbio.03020-24 (PMC11898617; doi:10.1128/mbio.03020-24)
Supplement: Supplemental material — Table S1 and Fig. S1-S5. [file mbio.03020-24-s0001.docx]

**Supporting Information**

**1. Supplementary tables**

**Table S1. Primers for qPCR**

|  | **Left primer (5'-3')** | **Right primer (5'-3')** |
| --- | --- | --- |
| *IL-1β* | GGTCAAAGGTTTGGAAGCAG | TGTGAAATGCCACCTTTTGA |
| *IL-6* | ACCAGAGGAAATTTTCAATAGGC | TGATGCACTTGCAGAAAACA |
| *TNF-α* | GGTGCCTATGTCTCAGCCTCTT | GCCATAGAACTGATGAGAGGGAG |
| *Cxcl2* | CATCCAGAGCTTGAGTGTGACG | GGCTTCAGGGTCAAGGCAAACT |
| *HnRNPa2b1* | CGGTGGCAATTTTGGACCAGGA | CCATAACCAGGGCTACCTCCAA |
| *pri-miR106b* | AAAGTGCTGACAGTGCAGA | GAACATGTCTGCGTATCTC |
| *pri-miR17* | AAGTGCTTACAGTGCAGGT | GAACATGTCTGCGTATCTC |
| *pri-miR324* | ATCCCCTAGGGCATTGGT | GAACATGTCTGCGTATCTC |
| *Nlrp3* | TCACAACTCGCCCAAGGAGGAA | AAGAGACCACGGCAGAAGCTAG |
| *Caspase1* | GGCACATTTCCAGGACTGACTG | GCAAGACGTGTACGAGTGGTTG |
| *18S* | AGTCCCTGCCCTTTGTACACA | CGATCCGAGGGCCTCACTA |

**2. Supplementary figures**


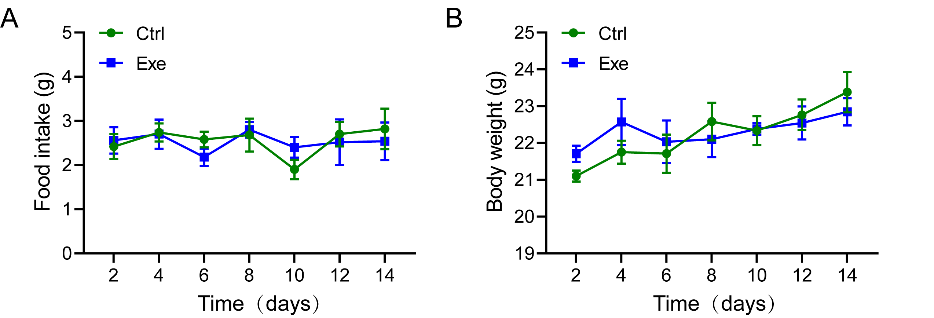


**Fig. S1.** The impact of Exe treatment on food intake and body weight in mice. **(**A) The food intake was monitored during Exe treatment. n=8. **(**B) The body weight was monitored during Exe treatment. n=8. All data are expressed as mean ± SEM.


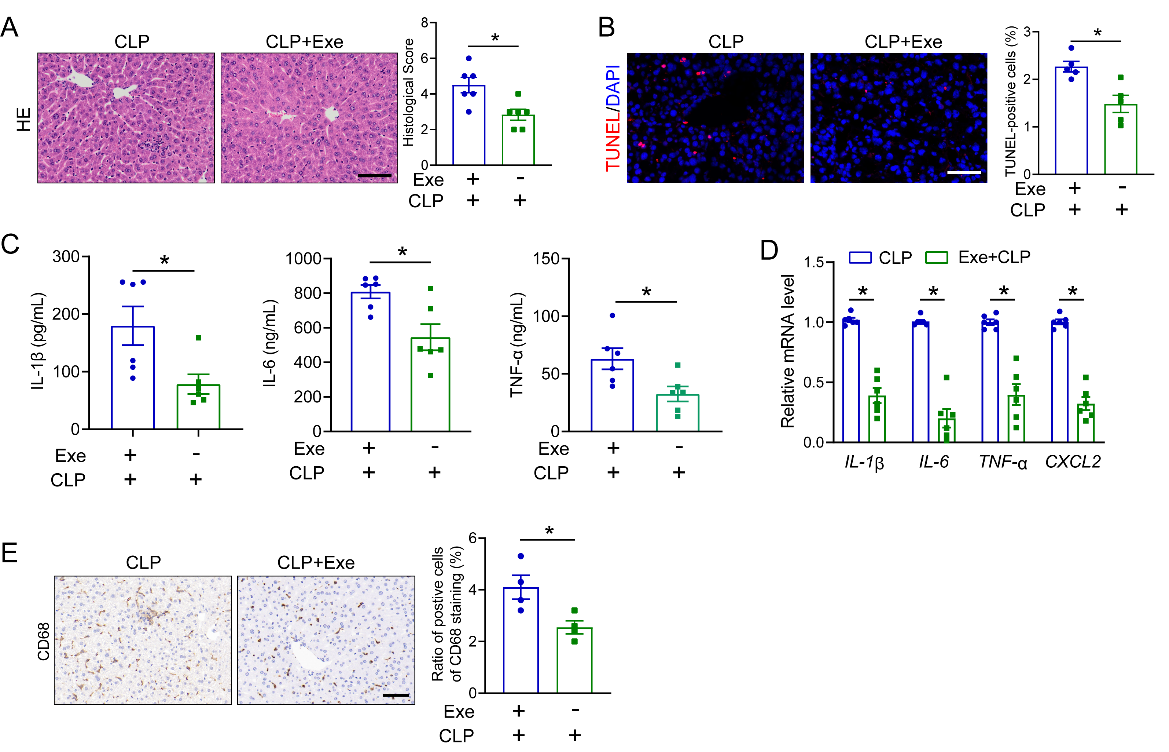


**Fig. S2.** The protective effect of Exe against SALI. **(**A) Representative H&E staining images and the histological score for lives in septic mice with or without Exe treatment, scale bar:100 µm, n=6. **(**B) TUNEL staining of the liver from septic mice with or without Exe treatment and quantification of dead cells, n = 5. **(**C) The levels of TNF-α, IL-1β, and IL-6 in plasma from septic mice with or without Exe treatment, n = 6. **(**D) The mRNA levels of IL-6, IL-1β, TNF-α, and Cxcl2 in the liver of septic mice with or without Exe treatment, n=6. **(**E) Immunochemistry staining and quantification of CD68^+^ cells in the liver sections from septic mice with or without Exe treatment, scale bar:100 µm, n=4. All data are expressed as mean ± SEM. **p*<0.05, as indicated.


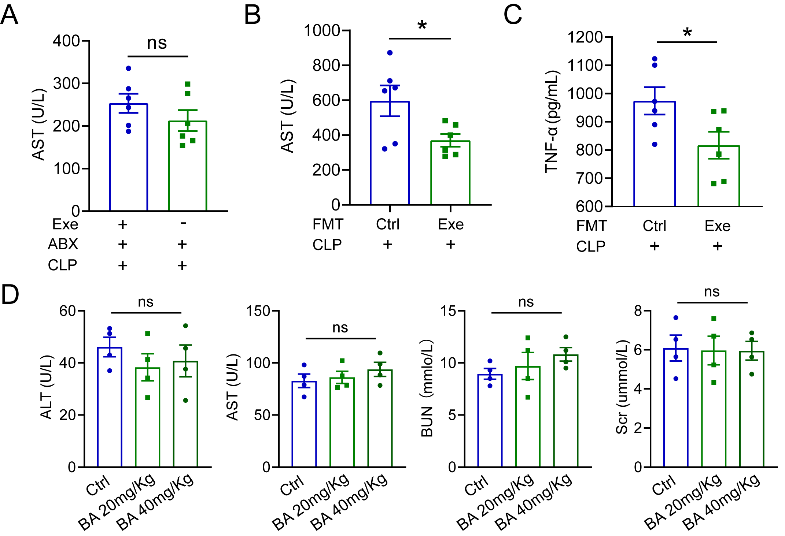


**Fig. S3. (**A) Plasma levels of AST from ABX-pretreated septic mice with or without Exe treatment, n=6. **(**B) Plasma levels of AST from septic mice received fecal suspension from Ctrl and Exe mice, n=6. **(**C) Plasma levels of TNF-α from septic mice received fecal suspension from Ctrl and Exe mice, n=6. (D) Plasma levels of ALT, AST, blood urea nitrogen (BUN), and serum creatinine (Scr) from mice with either 20 or 40 mg/kg BA treatment, n=4. All data are expressed as mean ± SEM. **p*< 0.05, ^ns^*p*>0.05, as indicated.


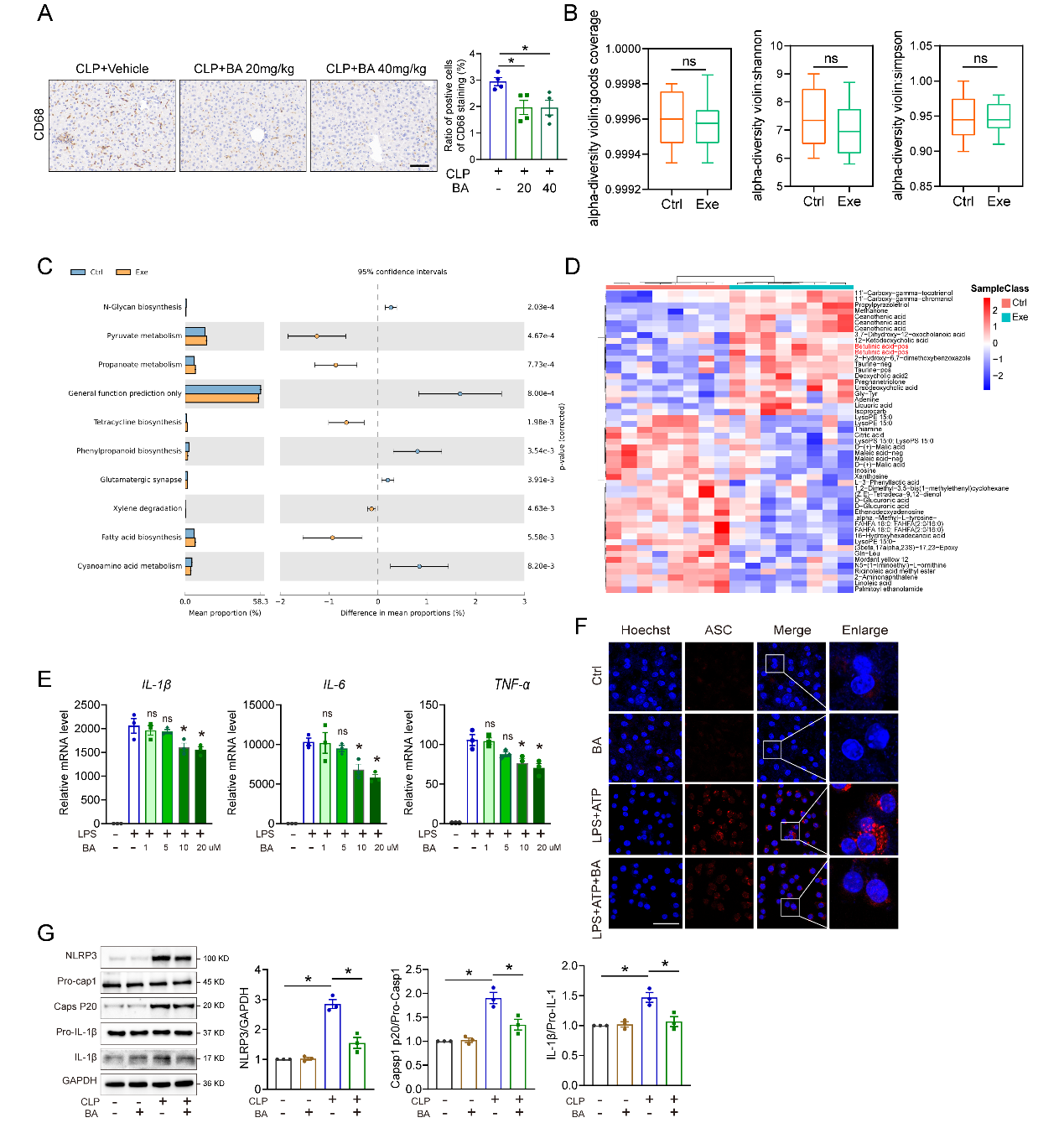


**Fig. S4. (**A) Immunohistological staining of CD68 in liver tissues and the quantification of CD68-positive stained cells, scale bar:100 µm, n=4. **(**B) Boxplots illustrating alpha diversity based on good coverage, Shannon index, and Simpson index in Ctrl and Exe-treated mice, n=8. **(**C) Microbiome functions were predicted based on operational taxonomic units (OTUs) using PICRUSt analysis in Ctrl and Exe-treated mice by PICRUSt analysis, n=8. (D) The differential metabolites of gut microbial in Ctrl and Exe-treated mice were visualized using a heat map, n=8. (E) The mRNA levels of IL-1β, IL-6, and TNF-α in BMDMs after LPS stimulation for 3 hours with or without BA (1, 5, 10, or 20µM), n=3. (F) Immunofluorescence staining of ASC oligomerization in BMDMs. (G) Expression of NLRP3 inflammasome-associated proteins NLRP3, Caps P20, and IL-1β in BMDMs by western blot, n=3. All data are expressed as mean ± SEM. **p*< 0.05, ^ns^*p*>0.05, as indicated.


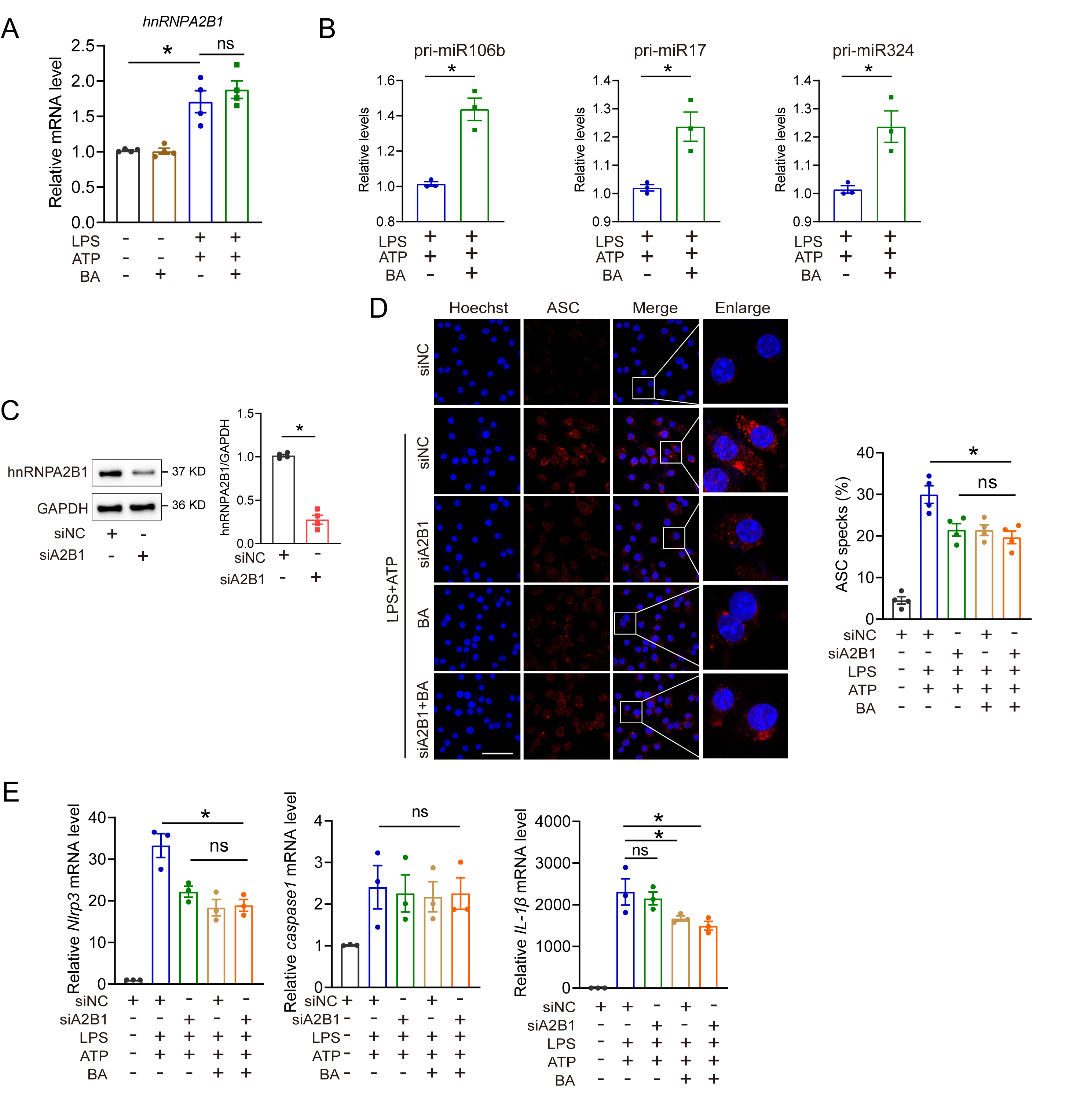


**Fig. S5. (**A) The mRNA level of *hnRNPA2B1* in BMDMs. After being primed with LPS and BA as described above, BMDMs were stimulated with 5 mM ATP for 30 minutes, n = 4. **(**B) The level of pri-miR106b, pri-miR17, and pri-miR324 in BMDMs. After being primed with LPS and BA as described above, BMDMs were stimulated with 5 mM ATP for 30 minutes, n = 4. **(**C) Knockdown of hnRNPA2B1 in BMDMs by siRNA, n = 3. **(**D) quantification of ASC oligomerization in BMDMs. BMDMs were subjected to a 36-hour transfection with siRNA specifically targeting hnRNPA2B1 or control siRNA. Following this, BMDMs were treated as described above, n = 4. (A) Relative mRNA expression of Nlrp3, Caspase1, and IL-1β in BMDMs. BMDMs were subjected to a 36-hour transfection with siRNA specifically targeting hnRNPA2B1 (siA2B1) or control siRNA (siNC). Following this, BMDMs were treated as described above, n =3. All data are expressed as mean ± SEM. **p*< 0.05, ^ns^*p*>0.05, as indicated.
